# Iatrogenic mitral chordal rupture induced by microaxial flow pump in acute myocardial infarction complicated by cardiogenic shock—a case report

**DOI:** 10.1093/ehjcr/ytaf550

**Published:** 2025-10-22

**Authors:** Tharusan Thevathasan, Ulf Landmesser, Stephan Jacobs, Carsten Skurk, Andi Rroku

**Affiliations:** Department of Cardiology, Angiology and Intensive Care Medicine, Deutsches Herzzentrum der Charité (DHZC), Campus Benjamin Franklin, Hindenburgdamm 30, Berlin 12203, Germany; Berlin Institute of Health, Anna-Louisa-Karsch-Strasse 2, 10178 Berlin, Germany; Deutsches Zentrum für Herz-Kreislauf-Forschung, Partner Seite Berlin, Potsdamer Str. 58, 10785 Berlin, Germany; Department of Cardiology, Angiology and Intensive Care Medicine, Deutsches Herzzentrum der Charité (DHZC), Campus Benjamin Franklin, Hindenburgdamm 30, Berlin 12203, Germany; Berlin Institute of Health, Anna-Louisa-Karsch-Strasse 2, 10178 Berlin, Germany; Deutsches Zentrum für Herz-Kreislauf-Forschung, Partner Seite Berlin, Potsdamer Str. 58, 10785 Berlin, Germany; Deutsches Zentrum für Herz-Kreislauf-Forschung, Partner Seite Berlin, Potsdamer Str. 58, 10785 Berlin, Germany; Department of Cardiothoracic and Vascular Surgery, Deutsches Herzzentrum der Charité (DHZC), Campus Virchow Klinikum, Berlin, Germany; Department of Cardiology, Angiology and Intensive Care Medicine, Deutsches Herzzentrum der Charité (DHZC), Campus Benjamin Franklin, Hindenburgdamm 30, Berlin 12203, Germany; Deutsches Zentrum für Herz-Kreislauf-Forschung, Partner Seite Berlin, Potsdamer Str. 58, 10785 Berlin, Germany; Department of Cardiology, Angiology and Intensive Care Medicine, Deutsches Herzzentrum der Charité (DHZC), Campus Benjamin Franklin, Hindenburgdamm 30, Berlin 12203, Germany; Deutsches Zentrum für Herz-Kreislauf-Forschung, Partner Seite Berlin, Potsdamer Str. 58, 10785 Berlin, Germany

**Keywords:** Cardiogenic shock, Microaxial flow pump, Mitral regurgitation, Chordal rupture, Mechanical circulatory support, Case report

## Abstract

**Background:**

Mechanical circulatory support devices, such as microaxial flow pumps (mAFP), are used in the management of cardiogenic shock, particularly in the setting of ST-segment elevation myocardial infarction. While these devices are potentially life-saving, their use is not without risk and might be associated with rare but serious complications, including structural cardiac injury.

**Case summary:**

We report the case of a 41-year-old male with known polysubstance abuse who experienced an out-of-hospital cardiac arrest due to ST-segment elevation myocardial infarction. Following successful return of spontaneous circulation and emergent percutaneous coronary intervention, a mAFP was implanted for haemodynamic stabilization. Echocardiographic monitoring initially confirmed correct positioning without evidence of valvular dysfunction. However, on Day 4, imaging revealed entrapment of the mAFP within the mitral valve’s subvalvular apparatus. Following careful device explantation, the patient showed new-onset severe mitral regurgitation (MR) due to posterior leaflet chordal rupture. Transoesophageal echocardiography confirmed a flail P3 segment. The presence of severe MR hindered adequate recompensation and complicated the weaning process from ventilatory support. The immediately consulted multidisciplinary Heart Team recommended early surgical intervention. The patient subsequently underwent successful mitral valve repair, resulting in clinical stabilization and eventual discharge to a rehabilitation facility in improved condition.

**Discussion:**

This case highlights a potential rare iatrogenic complication of mAFP support and emphasizes the necessity of meticulous echocardiographic surveillance, timely recognition of complications, and guideline-based multidisciplinary decision-making in the management of critically ill patients.

Learning points

- Structural valvular damage is a rare but important complication of microaxial flow pump support.
- Serial echocardiographic evaluation and routine monitoring of device position, function, and haemolysis markers are essential.
- A Heart Team approach ensures guideline-based and timely surgical intervention.

## Introduction

Mechanical circulatory support (MCS) systems, such as microaxial flow pumps (mAFPs), are increasingly employed for temporary haemodynamic stabilization in critically ill patients with myocardial infarction-related cardiogenic shock.^1^ mAFPs enable active left ventricular unloading and augmentation of cardiac output, offering a valuable bridge-to-recovery or bridge-to-decision strategy. Despite their growing use, these devices are not without risks.

A growing body of literature highlights the substantial incidence of complications associated with mAFP use in intensive care settings. These include major bleeding, vascular injury, haemolysis, and need for renal replacement therapy, as reported in larges studies.^1–3^ Additionally, device-related valvular trauma is recognized as a very rare but clinically significant complication, particularly affecting the mitral valve apparatus.^4^ The close anatomical proximity of the device’s inflow cannula to the subvalvular structures renders them vulnerable to mechanical stress, especially in small or underfilled ventricles of critically ill patients.

Importantly, such complications may remain subclinical or be misattributed to the underlying critical illness unless monitored with meticulous attention. Without regular imaging and laboratory surveillance, device malposition, progressive valve injury, or haemolysis can go undetected, potentially resulting in delayed diagnosis and adverse outcomes. We present a case of iatrogenic mitral chordal rupture in a patient supported with a mAFP following ST-segment myocardial infarction and cardiac arrest, underscoring the need for vigilance and multidisciplinary management in critically ill patients receiving MCS.^5^

## Summary figure

**Figure:**
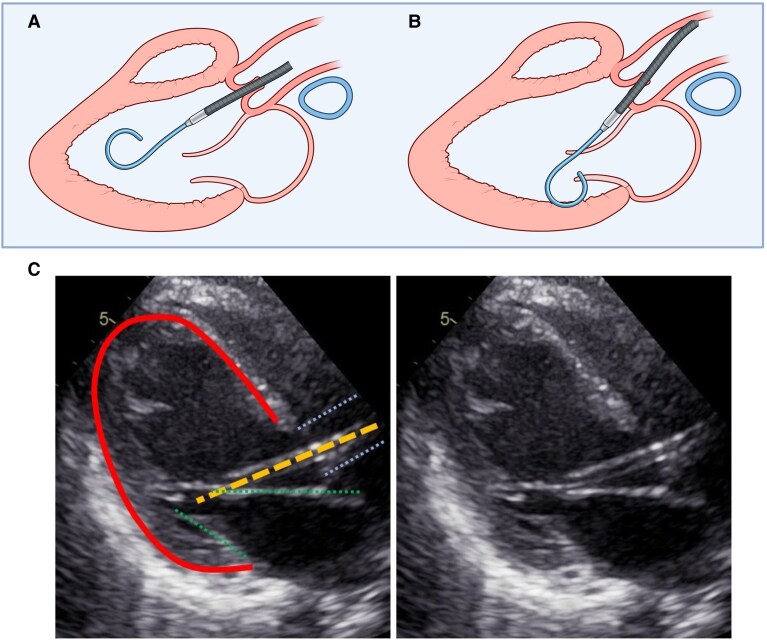
Malrotation and entrapment of a microaxial flow pump in the mitral valve apparatus: Schematic and echocardiographic correlation in a critically ill patient. (a) Schematic illustration of correct mAFP positioning with the inflow cannula oriented towards the left ventricular cavity and located 3.5–4.0 cm below the aortic valve. The device avoids direct contact with the mitral valve apparatus, enabling safe and effective left ventricular unloading. (b) Illustration of malrotated mAFP orientation with entrapment of the inflow cannula within the mitral valve subvalvular apparatus, as observed in the presented case. Malrotation alters the intended direction of flow and increases the risk of valvular injury. (c) Parasternal long-axis TTE images of the case. Left panel, An annotated TTE frame showing the left ventricular contour (red line), mitral valve leaflets (green lines), left ventricular outflow tract (light blue lines), and the entrapped mAFP cannula (yellow dashed line) course through the mitral valve apparatus. Right panel, The same TTE image without annotations for unaltered structural assessment. Abbreviations: mAFP, microaxial flow pump; TTE, transthoracic echocardiography.

## Case presentation

**Figure 1:**
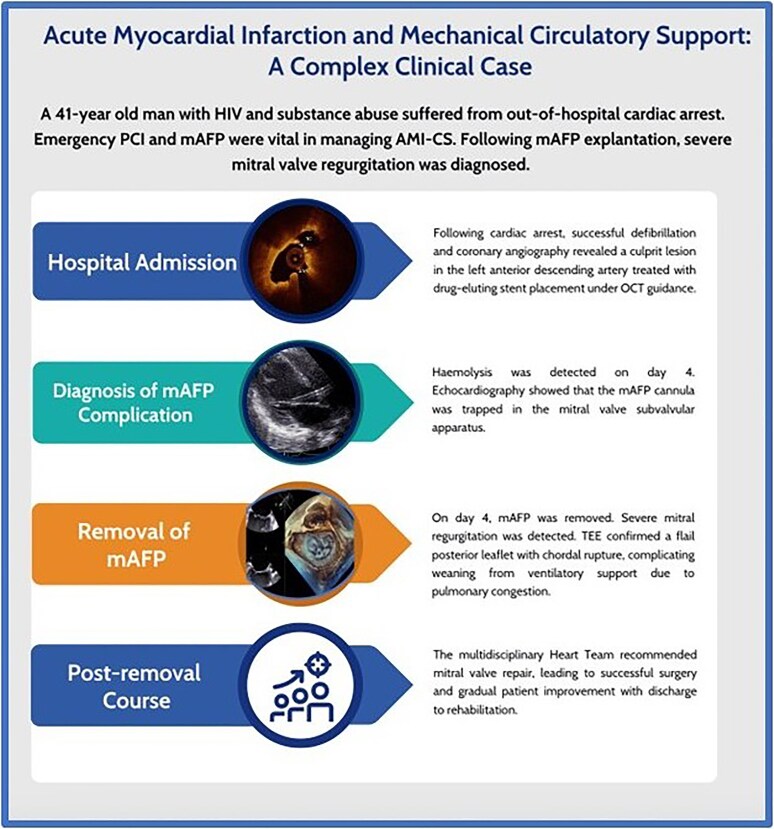
Timeline summary of acute myocardial infarction and microaxial flow pump-associated mitral valve complication in a critically ill patient. The infographic summarizes the sequence of clinical events in a 41-year-old man with HIV and substance abuse who suffered an out-of-hospital cardiac arrest. Following successful defibrillation and coronary revascularization with OCT-guided stent implantation, a mAFP was inserted for haemodynamic support due to cardiogenic shock. On Day 4, haemolysis was detected and echocardiography revealed entrapment of the mAFP within the mitral valve subvalvular apparatus. The device was subsequently removed and TEE confirmed severe mitral regurgitation caused by a flail posterior leaflet with chordal rupture. The multidisciplinary Heart Team recommended early surgical intervention. Mitral valve repair was successfully performed, allowing for gradual clinical improvement and discharge to rehabilitation. Abbreviations: AMI-CS, acute myocardial infarction-related cardiogenic shock; HIV, human immunodeficiency virus; mAFP, microaxial flow pump; OCT, optical coherence tomography; PCI, percutaneous coronary intervention; TEE, transoesophageal echocardiography.

A 41-year-old male with a history of tobacco use, well-controlled human immunodeficiency virus (HIV) under antiretroviral therapy and known substance abuse (cocaine, mephedrone, sildenafil, and nitrates) experienced an out-of-hospital cardiac arrest after acute dyspnoea. Bystander cardiopulmonary resuscitation was initiated immediately, followed by advanced life support and successful defibrillation for ventricular fibrillation. In light of persistent ST-segment elevations observed on the electrocardiogram after return of spontaneous circulation, the patient underwent emergency coronary angiography,^6^ which identified a culprit lesion in the proximal left anterior descending artery. A drug-eluting stent was implanted under optical coherence tomography guidance. Given ST-segment elevation myocardial infarction complicated by cardiogenic shock (characterized by a left ventricular ejection fraction of approximately 30% and an elevated left ventricular end-diastolic pressure of 37 mmHg), a mAFP was subsequently inserted for haemodynamic support, as described in a previous trial (*Figure 1* for clinical course).^1^

**Figure 2:**
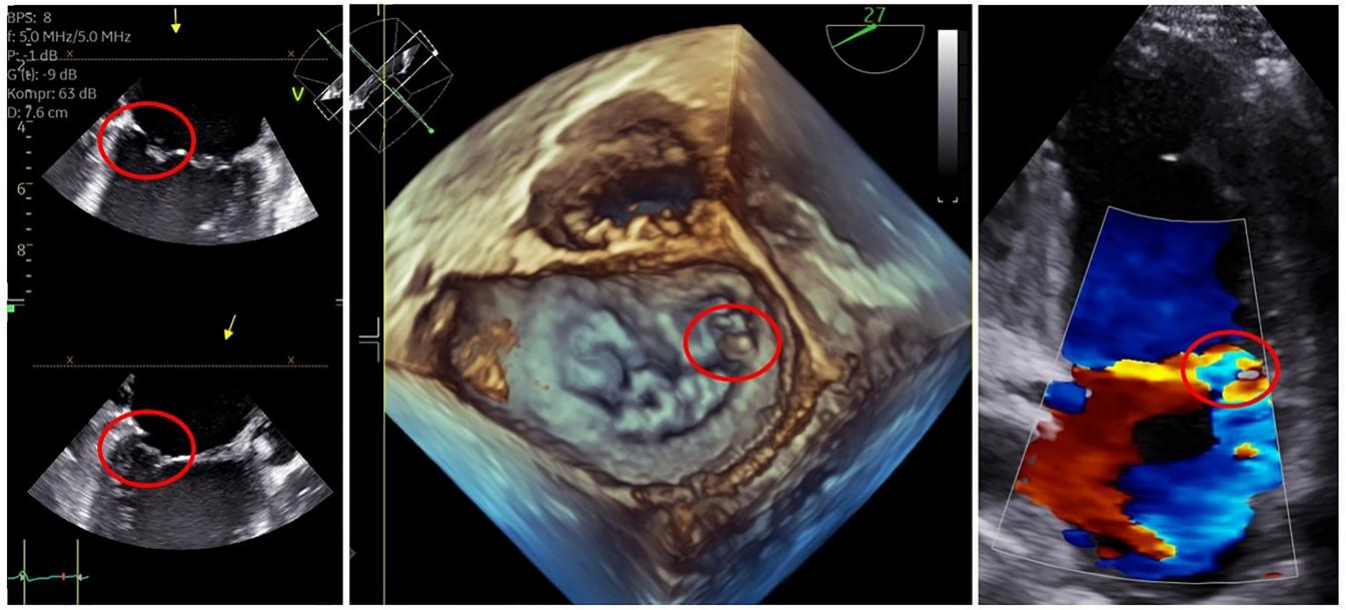
Multimodal transoesophageal echocardiographic visualization of flail posterior mitral leaflet with chordal rupture following microaxial flow pump support. Two-dimensional (left and right panels) and three-dimensional (middle panel) transoesophageal echocardiographic images obtained after explantation of the microaxial flow pump, demonstrating flail posterior mitral leaflet (PML) with chordal rupture. The rupture site is highlighted by circles across all modalities. The middle panel presents an *en face* 3D view of the mitral valve from the left atrial perspective, clearly delineating the disrupted subvalvular structure. The right panel, using colour Doppler imaging, visualizes the associated severe mitral regurgitation.

Daily transthoracic echocardiographic (TTE) monitoring was initiated following mAFP implantation to assess device positioning and detect any evolving complications. Initial echocardiography showed that the inlet opening of the mAFP was positioned at the mid-ventricular level, which typically corresponds to a location ∼3.5–4 cm distal to the aortic valve. No valvular pathology was detected. By Day 4, laboratory surveillance indicated the onset of haemolysis, evidenced by rising lactate dehydrogenase, elevated free haemoglobin, and declining haptoglobin levels. Echocardiography revealed that the mAFP cannula was entrapped within the subvalvular apparatus of the mitral valve (Summary figure). Despite this, the device console did not trigger any alarms indicative of suction or malposition, and haemodynamic support remained stable at that time. Given clinical haemodynamic stabilization on the same day, the mAFP was weaned to P2 support level and carefully explanted. Post-removal echocardiography revealed new-onset severe mitral regurgitation. Transoesophageal echocardiography (TEE) confirmed a flail posterior leaflet (P3 to P2) with chordal rupture (*Figure 2* and Supplementary material online, *Videos S1*  to  *S3*). Due to the severity of the mitral regurgitation, haemodynamic stabilization and recompensation was challenging. The patient required prolonged mechanical ventilation and weaning attempts from ventilatory support were impaired by the underlying volume overload and pulmonary congestion.

The case was promptly evaluated by the multidisciplinary Heart Team, who advocated for timely surgical intervention. Consequently, the patient underwent a successful mitral valve repair. After the surgery, the patient improved gradually and was discharged to rehabilitation.

## Discussion

This case exemplifies a very rare but clinically significant complication of mAFP use—iatrogenic mitral chordal rupture. Mechanical interference with the mitral subvalvular apparatus, particularly in small or hyperdynamic ventricles, remains a plausible mechanism of injury.^4^ The inflow cannula, when malpositioned or misoriented, may interact with delicate subvalvular structures, such as the chordae tendineae or the posterior mitral leaflet, causing progressive trauma. The continuous suction effect and rotational forces generated by the pump’s impeller—especially when the device is in close proximity to the mitral apparatus—can result in cumulative structural stress. Even transient periods of suction or minor displacements may suffice to weaken and ultimately rupture valvular elements. This risk is further heightened in settings of reduced ventricular dimensions, where spatial tolerance is limited.

Recent evidence highlights that correct insertion depth alone is insufficient to ensure optimal positioning.^7^ In a cohort of patients with cardiogenic shock, malrotation of the mAFP device—defined as improper angular orientation of the inflow cannula towards the mitral valve despite correct depth—was observed in one-third of patients and was significantly associated with worsening mitral regurgitation, aortic regurgitation, and adverse in-hospital events including ischaemic stroke and major bleeding.^7^ Notably, conventional fluoroscopic views and console pressure tracings failed to detect malrotation in these cases, underscoring the critical role of echocardiographic confirmation. Echocardiography thus remains a reliable modality to detect both axial and rotational malposition and should be integrated as standard-of-care during and after device insertion.

Daily echocardiographic surveillance, with precise assessment of inflow and outflow positioning relative to the aortic and mitral valves, is essential. International recommendations advise maintaining the inflow 3.5–4.0 cm below the aortic annulus and directed away from the mitral apparatus, while the outflow should remain proximal to the aortic valve.^8,9^ In this case, serial imaging allowed for timely identification of a new flail posterior mitral leaflet with severe regurgitation as an explanation for the lack of clinical improvement. Early recognition facilitated prompt multidisciplinary decision-making, leading to successful surgical valve repair.

In parallel with clinical and imaging assessment, laboratory monitoring remains essential for early identification of device-related complications. Elevations in haemolysis markers may indicate mAFP misplacement due to shear stress or mechanical contact with valvular or endocardial structures. These laboratory derangements may precede clinical deterioration and should prompt immediate device reassessment. The presence of device-induced valvular dysfunction may further complicate this balance by increasing turbulence.

In addition, this case reinforces the importance of individualized Heart Team-based decision-making. According to the European Society of Cardiology guidelines, patients with acute severe mitral regurgitation and a high likelihood of successful valve repair should undergo urgent surgery (Class I, Level B).^10^ Adherence to this guidance in our patient enabled timely valve repair facilitating clinical stabilization before the development of ventricular remodelling or multi-organ failure.

In summary, mAFP support can be life-saving in selected patients with cardiogenic shock but carries intrinsic risks that demand rigorous monitoring. Malrotation within the left ventricle is an underrecognized but not uncommon phenomenon that can significantly impair unloading efficiency and precipitate structural damage. Accurate detection requires systematic echocardiographic imaging beyond standard console diagnostics. As this case illustrates, close integration of advanced imaging, biochemical surveillance and multidisciplinary expertise is vital to mitigating risk and achieving favourable outcomes in patients supported with percutaneous MCS.

## Supplementary Material

ytaf550_Supplementary_Data

## Data Availability

All relevant clinical data are presented within the article. Further anonymized data can be made available upon reasonable request to the corresponding author.

## Lead author biography

Dr. Tharusan Thevathasan is a physician in the Department of Cardiology, Angiology and Intensive Care Medicine and research coordinator at the Berlin Cardiac Arrest Center of the Deutsches Herzzentrum der Charit (Campus Benjamin Franklin). His clinical and academic focus is on cardiogenic shock, refractory cardiac arrest, and mechanical circulatory support. Dr. Thevathasan has conducted research in critical care medicine at leading institutions including Massachusetts General Hospital/Harvard Medical School, University College London and Oxford University Hospitals. He is dedicated to advancing patient-centred care and interdisciplinary research in acute cardiovascular emergencies.
